# CHFR promotes metastasis of human gastric carcinoma by activating AKT and ERK via NRF2- ROS axis

**DOI:** 10.1186/s12876-023-02724-4

**Published:** 2023-04-06

**Authors:** Feiyun He, Bin Ye, Xiaomeng Wu, Jundi Pan, Jianbo Wang, Xiaojing Wang

**Affiliations:** 1 Department of Gastroenterology, Lishui Chinese Medicine Hospital, Lishui, 323000 Zhejiang Province China; 2 Department of Gastroenterology, The Fifth Affiliated Hospital of Wenzhou Medical University and Lishui Municipal Central Hospital, Lishui, 323000 Zhejiang Province China; 3 Wenzhou Medical University, Wenzhou, 325035 Zhejiang Province China

**Keywords:** Gastric cancer, CHFR, Metastasis, NRF2, ROS, AKT, ERK

## Abstract

Tumor suppressor gene CHFR (The Checkpoint with Forkhead-associated and Ring finger domains) is a mitotic checkpoint and frequently hypermethylated in gastric cancer. Our previous study found CHFR played a certain extent pro-tumor function in gastric cancer. However, little is known about the underlying mechanism. In this study, we tried to further elucidate the role and mechanism for CHFR in gastric cancer (GC) by constructing CHFR stably expressed cell lines. As expected, the ectopic expression of CHFR slowed the cell proliferation in both two SGC-7901 and AGS cells, while significantly promoted the potential of cell migration and invasion. For the first time, our data indicated that stable expression of CHFR in SGC-7901 and AGS restrained cellular reactive oxygen species (ROS) generation and promoted the activation of AKT and ERK, two regulators of redox hemostasis. Furthermore, H_2_O_2_ treatment effectively elevated ROS level and reversed CHFR-induced cell invasion in stable SGC-7901 and AGS cells with the decreased phosphorylation of AKT and ERK. We also confirmed that CHFR exerted its function by promoting NRF2 expression. The most important is, the ectopic expression of CHFR significantly inhibited SGC-7901 cell-derived xenografts and obviously promoted lung metastasis of GC cell with NRF2, p-AKT and p-ERK increased. Taken together, our findings suggested that CHFR might take part in gastric cancer progression especially cancer metastasis by activating AKT and ERK via NRF2- ROS axis.

**Supplementary Information:**

The online version contains supplementary material available at 10.1186/s12876-023-02724-4.

## Introduction

Gastric cancer is the third leading cause of cancer-correlated death in both sexes worldwide, with a high morbidity and mortality in China [1–3]. In the recent decades, the incidence rate trend of GC is declining. However, there is still about one million new GC patients are diagnosed annually [4]. Due to the recurrence and metastasis of advanced GC, the prognosis for large portion of patients remains relatively poor. Therefore, it is gratified to further illustrate the mechanisms at molecular level for therapeutic targets development for the treatment of GC, especially metastatic ones.

CHFR, a protein containing forkhead-associated and ring finger domains, plays as a checkpoint to take part in cell-cycle regulation by delaying entrance the metaphase in response to microtubule stress [5]. Multiple studies have indicated that CHFR functioned as a tumor suppressor by regulating numerous important proteins as it was also a RING-type E3 ubiquitin (Ub)-ligase [6, 7]. The expression of *CHFR* was found to be downregulated by promoter methylation or mutated in various cancers such as colorectal cancer [8], esophageal cancer [9], human non-small-cell lung cancer [10], GC [11]. Our previous research reported that CHFR not only a regulator for cell cycle progression, but also promoted GC cell migration and invasion in vivo [12]. However, the biological role and its underlying mechanism of CHFR in cancer, especially in GC were still extremely limited documented up to now.

In the present study, CHFR stably overexpressed GC cell lines were constructed and we explored the function of CHFR in GC both in vitro and in vivo. Our data indicated that CHFR indeed suppressed cell cycle progression of GC cells, but enhance their metastasis potential. Mechanistically, we proved that CHFR could elevated the anti-oxidant modulator NRF2, and decrease the ROS levels, and activate the AKT and ERK signaling pathways. All these findings provided a novel insight into the role and underlying mechanism of CHFR in GC.

## Materials and methods

### Cell culture and CHFR over-expression stable cell line

The human gastric cancer cell lines AGS (derived from the stomach tissue of a 54-year-old female patient with gastric adenocarcinoma ) and SGC-7901 (isolated from the metastasis of untreated gastric adenocarcinoma of a 56-year-old female patient) were purchased from the American Type Culture Collection (ATCC, Manassas, VA, USA) and grown at 37 °C in Dulbecco’s Modified Eagle’s Medium (DMEM medium, Hyclone, Logan, Utah, USA, SH30081.02) supplemented with 10% fetal bovine serum (FBS, Gibco, California, USA, 10,091,148), 2 mM L-glutamine (Invitrogen, Carlsbad, California USA, 21,051,040),1% penicillin (100 units/ml) and streptomycin (100 µg/ml) (Invitrogen, Carlsbad, California USA, 15,140,122).

For stable overexpression of CHFR in AGS and SGC-7901 cells, the CHFR cDNA was amplified by PCR and subcloned into the LV-13 (pLenti-EF1a-LUC-F2A-Puro-CMV) vector for lentivirus package (GenePharma). AGS and SGC-7901 cells were infected with the concentrated virus with CHFR overexpression vector or empty vector. Subsequently, cells were treated with 2 µg/ml puromycin for 2 weeks to select cells with stable expression of CHFR, and the expression efficiency was validated by western blot analysis.

### Western blotting

Cells were lysed in radioimmunoprecipitation assay buffer (RIPA buffer) adding 1% protease inhibitor cocktail (Sigma, St. Louis,Missouri, USA, P8340). After electrophoresis on 12% or15% SDS–PAGE gels for 60 min at 200 V, proteins were then transferred onto polyvinylidene difluoride (PVDF) membranes. The PVDF membranes were blocked with 5% fat-free milk for 2 h at room temperature and incubated with primary antibodies against at 4℃ overnight. The corresponding horseradish peroxidase (HRP)-conjugated secondary antibody was added and incubated at room temperature for 1 h. After chemiluminescence reaction with HRP substrate, the signals were visualized. The primary antibodies against CHFR, AKT, p-AKT, ERK, p-ERK, and NRF2 were purchased from CST (Cell Signaling Technology, Boston, Massachusetts, USA, 6904 S, 4685 S, 4060 S, 4695T, 4370T, 12721T) and used at dilution 1:1000. The antibody against β-actin were purchased from Sigma (Sigma, St. Louis,Missouri, USA, A5441) and used at dilution at 1:5000.

### MTT assay for cell proliferation

In brief, the Lenti-control and Lenti-CHFR AGS and SGC-7901 cells were seeded into 96-well plates at the density of 5000 cells/well and incubated for 24 h, 48 h. After that, 50 µl MTT (chemically 3-(4,5-dimethylthiazol-2-yl)-2,5-diphenyltetrazolium bromide) solution in phosphate-buffered saline (PBS, 1 mg/ml) was added into each well and incubated for 2–4 h, and then 150 µl dimethyl sulfoxide (DMSO) was added to resolved the violet crystal. After shaking, a microplate reader (Molecular Device, Thermo Scientific, Waltham, Massachusetts, USA, 51,119,570) was using to read the absorbance of each sample at 570 nm.

### Flow cytometry assay for cell proliferation

Flow cytometry experiment was performed to examine cell proliferation using CSFE (Beyotime, Shanghai, China) staining according to previous publication [13]. In brief, cells were transfected with pcDNA3.1 vector or pcDNA3.1-CHFR overexpression plasmid with lipofectamine 2000 for 24 h, Then, cells were stained with CFSE (10 µM, diluted with serum-free medium) and incubated at 37℃for 15–30 min in dark. After that, cells were corrected by centrifuge and washed with PBS. Analyze was performed on flow cytometer (Thermo Scientific, Waltham, Massachusetts, USA, AttuneNxT), CFSE should be excited by the 488 nm laser and should be detected at 518 nm.

### Transwell assays for cell migration and invasion in vitro

Transwell assays were used to determine cell migration and invasion as previous study [14]. For the cell migration, a transwell system (24 wells, 8 μm pore size with poly-carbonate membrane, Corning Life Science, Corning, New York, USA,3422) were chose. The cells were suspended in serum-free DMEM medium and seeded into the upper chambers at the density of 30,000 cells/well. The chamber was then placed into 24 cell plate which was filled with 500 µl DMEM medium with 20% FBS as a chemoattractant. After incubation for 24 h, the cells remaining in the upper chamber were wiped, and the cells at the bottom of the chamber were fixed with iced methanol, stained in 0.5% crystal violet for 30 min at room temperature and counted under a microscope (Olympus Corp., Tokyo, Japan, CKX53). The results were averaged over three independent experiments.

For the cell invasion, all procedure was similar besides the transwell membrane was pre-coated with matrigel (BD Biocoat, Franklin Lake, New Jersey, USA,356,234).

### Assessment of ROS generation (Fluorometry and fluorescence microscope)

For ROS detection by fuorometry, harvest the CHFR stably expressed cells or control cells, and ensure a single cell suspension obtained by gently pipetting up and down suspension cells. After washing with PBS twice, cells were stained with 10 µM DCFDA (MedChemexpress, Monmouth Junction, New Jersey, USA, HY-D0940) and incubate for 30 min at 37 °C in dark. Once the incubation is completed, do not wash the cells. Analyze was performed on flow cytometer (Thermo Scientific, Waltham, Massachusetts, USA, AttuneNxT), DCFDA should be excited by the 488 nm laser and should be detected at 535 nm.

For ROS detection by fluorescence microscope, cell in the culture dish were washed with PBS twice, cells were stained with 20 µM DCFDA and incubate for 30 min at 37 °C in dark. Once the incubation is completed, do not wash the cells, and pictures was obtained under fluorescence microscope (Olympus Corp., Tokyo, Japan, CKX53).

### NRF2 silence

To knockdown the expression of NRF2, Small interfering RNA (siRNA) against NRF2 was purchased from GemmaPharma (Shanghai, China). siRNA against NRF2 or negative control (NC) was transfected with lipofectamine 3000 according to the manufactory’ instruction. The sequence for siRNA against NRF2 is 5’- UCCCGUUUGUAGAUGACAA-3’, and the sequence against NC is 5’-UUCUCCGAACGU GUCACGUTT-3’.

### Xenograft experiment

5*10 ^6^ CHFR stably expressed AGS cells and the control cells was subcutaneously injected into the right flank in Balb/c nude mice (female, 4 weeks, 20 g; Vital River Laboratories, China) to establish xenografts model. Each group contained right mice. Tumor size was obtained by a micrometer caliper. Tumor volume (mm³) was calculated using the following formula: V = (a×b2)/2. After largest tumors reached the volume of about 1000mm3, mice were killed after anesthesia with pentobarbital (50 mg/kg weight) and tumors were excised and weighed. All animal studies were done in compliance with the regulations and guidelines of Institutional Animal Care and Use Committee of Wenzhou Medical University, and conducted according to the Association for Assessment and Accreditation of Laboratory Animal Care International (AAALAC) and the Institutional Animal Care and Use Committees (IACUC) guidelines.

### Lung metastasis experiment

The lung metastasis model in nude mice was established as reported as previously publication [15]. In brief, 3*10^6^ CHFR stably overexpressed AGS cells and control cells were injected via tail vein. Each group contained six mice. After three weeks, the mice were treated with pentobarbital (50 mg/kg weight) and euthanized according with the approved guidelines and on the basis of an approved protocol by the Institutional Animal Care and Use Committee of Wenzhou Medical University and conducted according to the AAALAC and IACUC guidelines. Lungs were got out integrally and photographed. The tissues were used to perform the hematoxylin and eosin staining (HE staining) to assess the AGS cell metastasis in lung.

### Immunohistochemistry (IHC) staining

For IHC, tumors were fixed and prepared the sections with 8 μm thickness. After de-paraffinized and rehydration in graded ethanol, slides were immersed in 0.01 M citrate buffer, pH 6.0, using a steamer at 95℃ for antigen retrieval. Subsequently, sections were incubated with primary antibody (Ki67 (Cell Signaling Technology, Boston, Massachusetts, USA, 9449 S), NRF2 (Abcam, Cambridge, UK, ab62352), p-AKT (Cell Signaling Technology, Boston, Massachusetts, USA, 4060 S) and p-ERK (Cell Signaling Technology, Boston, Massachusetts, USA,4370T), all antibodies were diluted at 1:100 in BSA,) at 4 ℃ at least 4 h in a humidifying box. After washing with PBST, slides were incubated with the corresponding horseradish peroxidase–conjugated second antibodies for 2 h at room temperature. Finally, diaminobenzidene substrate was used for color development and counterstained with hematoxylin.

### Statistical and dataset analysis

All data are expressed as the means ± S.D. from at least 3 independent experiments. All statistical analyses were performed using GraphPad Prism 5.0 (GraphPad Software, Inc., La Jolla, CA, USA). The significantly statistical analysis was decided by using two-sided Student’s t test for two groups or one-way ANOVA for multiple groups. P < 0.05 (*) was considered significant.

## Results

### CHFR suppressed cell proliferation but promoted cell migration and invasion in gastric cancer

**Fig. 1:**
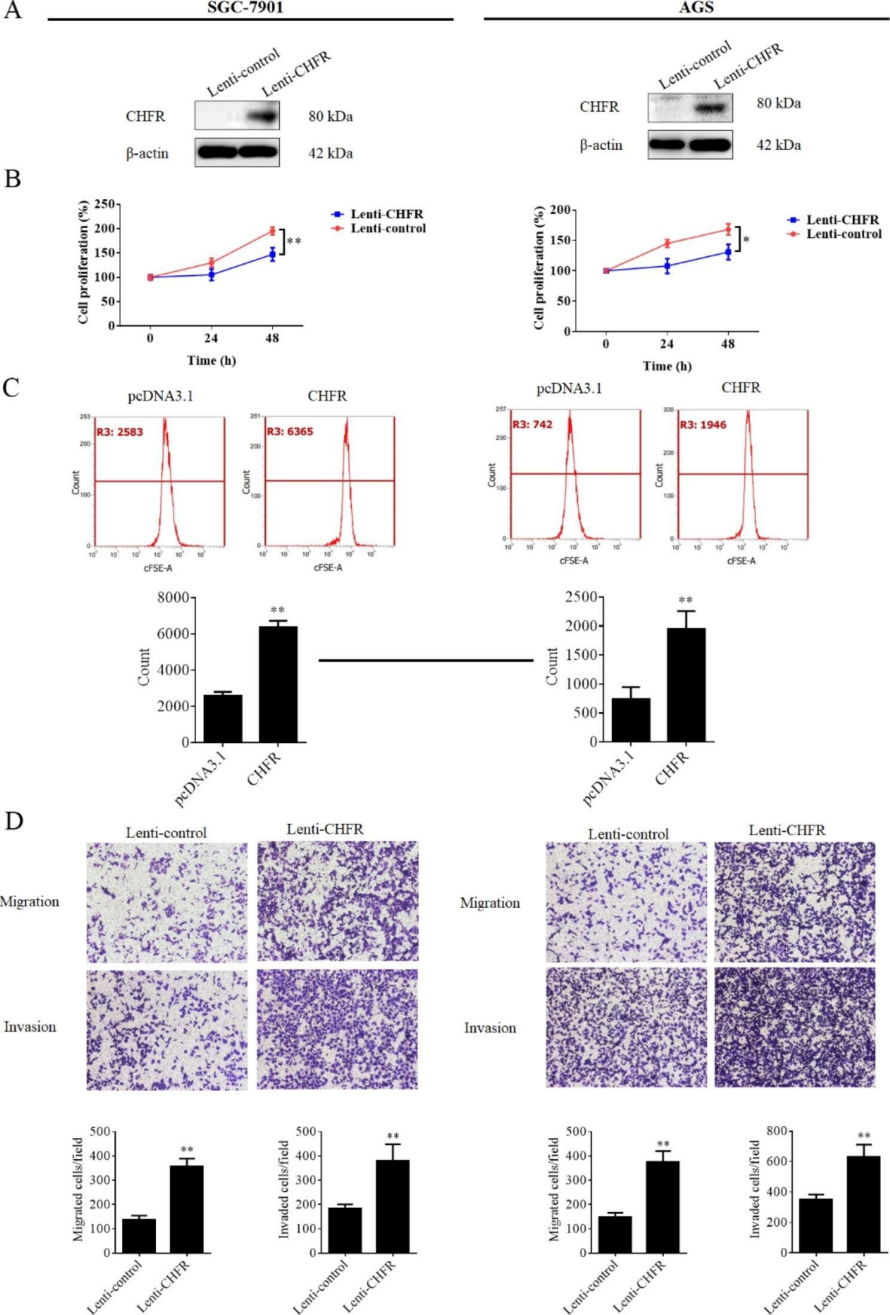
Overexpression of CHFR inhibited cell proliferation, but promoted cell migration and invasion of gastric cancer cells. (**A**) Western blot assay was used to determine the CHFR expression in gastric cancer cell lines transfected with CHFR overexpression lentivirus or the control. (**B**) MTT experiments was performed to assess the cell proliferation of gastric cancer cells with CHFR overexpressed or control cells. (**C**) Flow cytometry was used to detect cell proliferation of gastric cancer cell with CHFR overexpressed or control cells. (**D**)Transwell assay was chose to detect the cell migration and invasion potentials of SGC-7901 and AGS cells transfected with CHFR overexpression lentivirus and control lentivirus. Quantitative analysis was shown in the histogram. The data are derived from one of the three independent experiments. *P < 0.05, **P < 0.01

As mentioned above, CHFR was considered as a tumor suppressor in many cancer types. However, our published research indicated that there is a negative relevance between CHFR expression level and overall survival rate of gastric cancer patients, and enhanced the cell migration and invasion of GC cells [12]. This result suggest CHFR might be not a pure tumor suppressor at least in gastric cancer. To further verify the biological roles of CHFR in gastric cancer, CHFR stably expressed AGS and SGC-7901 cells were constructed using lentivirus and the expression efficiency was validated by western blot. As shown in Fig. 1A, the data revealed that protein levels of CHFR were significantly increased in SGC-7901 and AGS cells in Lenti-CHFR groups compared with those in control cells. As CHFR is a mitotic checkpoint which contributed for the regulating on cell cycle progression, we firstly detected the cell proliferation of two cell lines. As expected, the ectopic expression of CHFR significantly inhibited cell growth in both two cell lines (Fig. 1B). Similar results were obtained from flow cytometry experiments in which CSFE was used (Fig. 1C). This finding proved that exogenous CHFR exerted its normal biological functions in GC. Subsequently, transwell assays were performed to determine the cell migration and invasion in CHFR stably expressed cell lines. As shown in Fig. 1D, CHFR effectively promoted the cell migration and invasion in both two cell lines. These findings were in consistent with our previous results in which transient transfection was used [12].

### CHFR restrained cellular ROS generation in gastric cancer

**Fig. 2:**
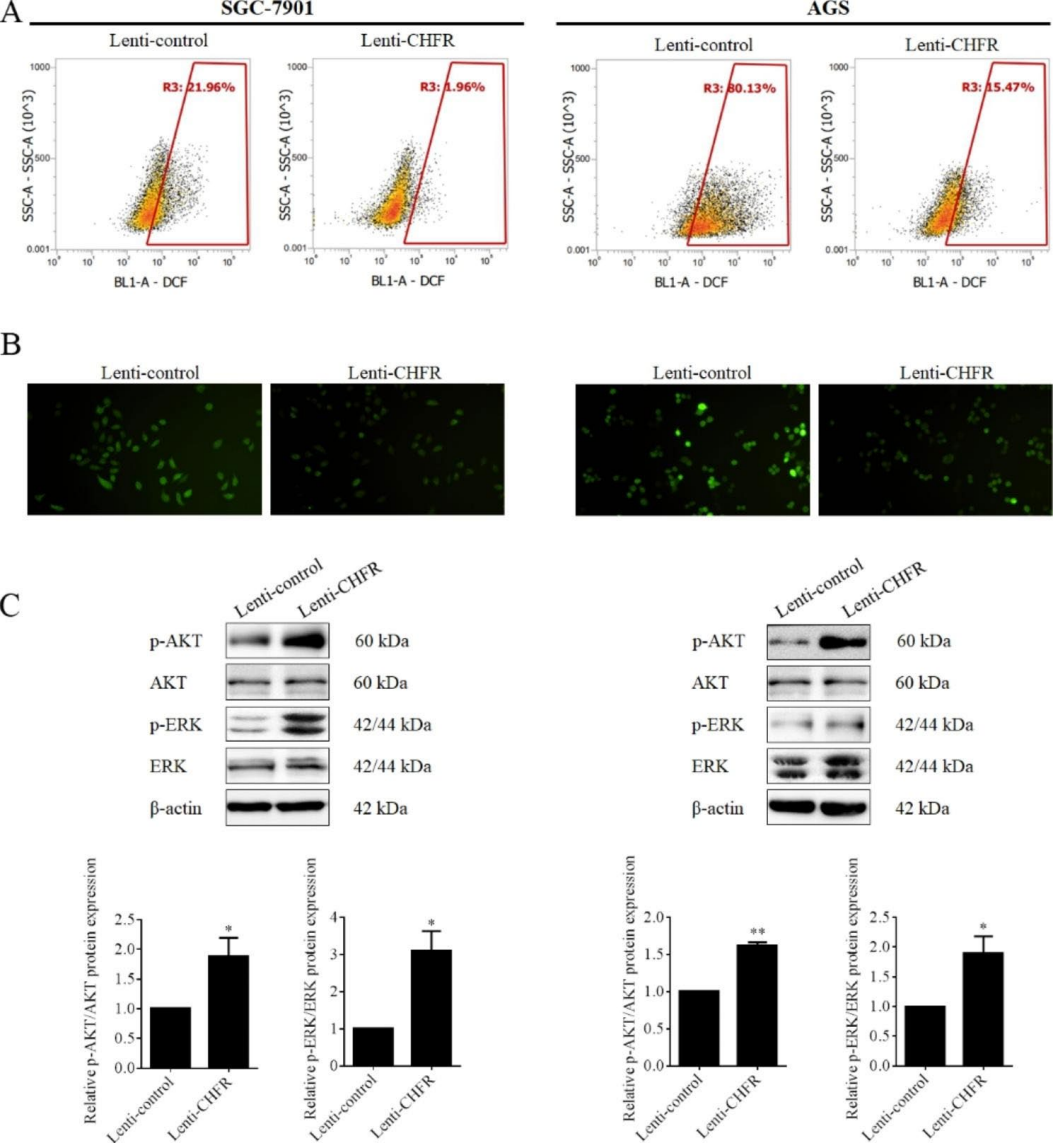
CHFR overexpression restrained cellular ROS generation and promoted AKT and ERK activation in gastric cancer cells. (**A**) The ROS level in SGC-7901 and AGS cells with stable CHFR expression were analyzed by flow cytometer and represented data were shown. (**B**) Representative fluorescence images of cellular ROS in stable SGC-7901 and AGS cells were presented. The data are derived from one of the three independent experiments. Western blot assay was used to determine the CHFR expression in gastric cancer cell lines transfected with CHFR overexpression lentivirus or the control. (**C**) Western blot assay was used to determine the phosphorylation of AKT and ERK in CHFR stably expressed gastric cancer cell lines or the control. Quantitative analysis expression of proteins was shown in the histogram. Data was presented by mean ± SD for three separate experiments. * P < 0.05, **P < 0.01

Although dozens of studies focusing on CHFR have been published, little is known about its roles and the underlying mechanisms in progression of cancers including gastric cancer. Our previous data also unveiled that CHFR could promoted cell migration and invasion of gastric cancer cells in which CHFR was transiently overexpressed using plasmid, but leaving the molecular mechanism to be further documented [12]. Interestingly, an earlier study reported that CHFR could negatively regulate the activity of SIRT1 by promoting its degradation upon oxidant stress [16]. Therefore, we next examined whether CHFR could take part in the regulation of ROS generation in gastric cancer. For the first time, our data revealed ROS levels in CHFR stably expressed SGC-7901 and AGS cells were much lower than those in their control cells (Fig. 2A and B).

It is well known that aberrant activation of PI3K/Akt signaling pathway contributed to increased ROS levels in cancer cells by driving many of the molecular mechanisms through direct modulation of mitochondrial bioenergetics and activation of NADPH oxidases (NOXs), or indirectly through the production of ROS as a metabolic by-product [17]. Therefore, we firstly examined the activation of AKT under CHFR overexpression in gastric cancer cells and unexpectedly the result indicated that the ectopic expression of CHFR significantly elevated the phosphorylation of AKT in SGC-7901 and AGS cells (Fig. 2C). Studies also demonstrated that the MAPK signal-regulated kinase ERK could be activated in response to oxidative stress [18]. Next, the activation of ERK in CHFR stably expressed gastric cancer cells were detected. As shown in Fig. 2C, overexpression of CHFR obviously promoted the phosphorylation of ERK.

**Fig. 3:**
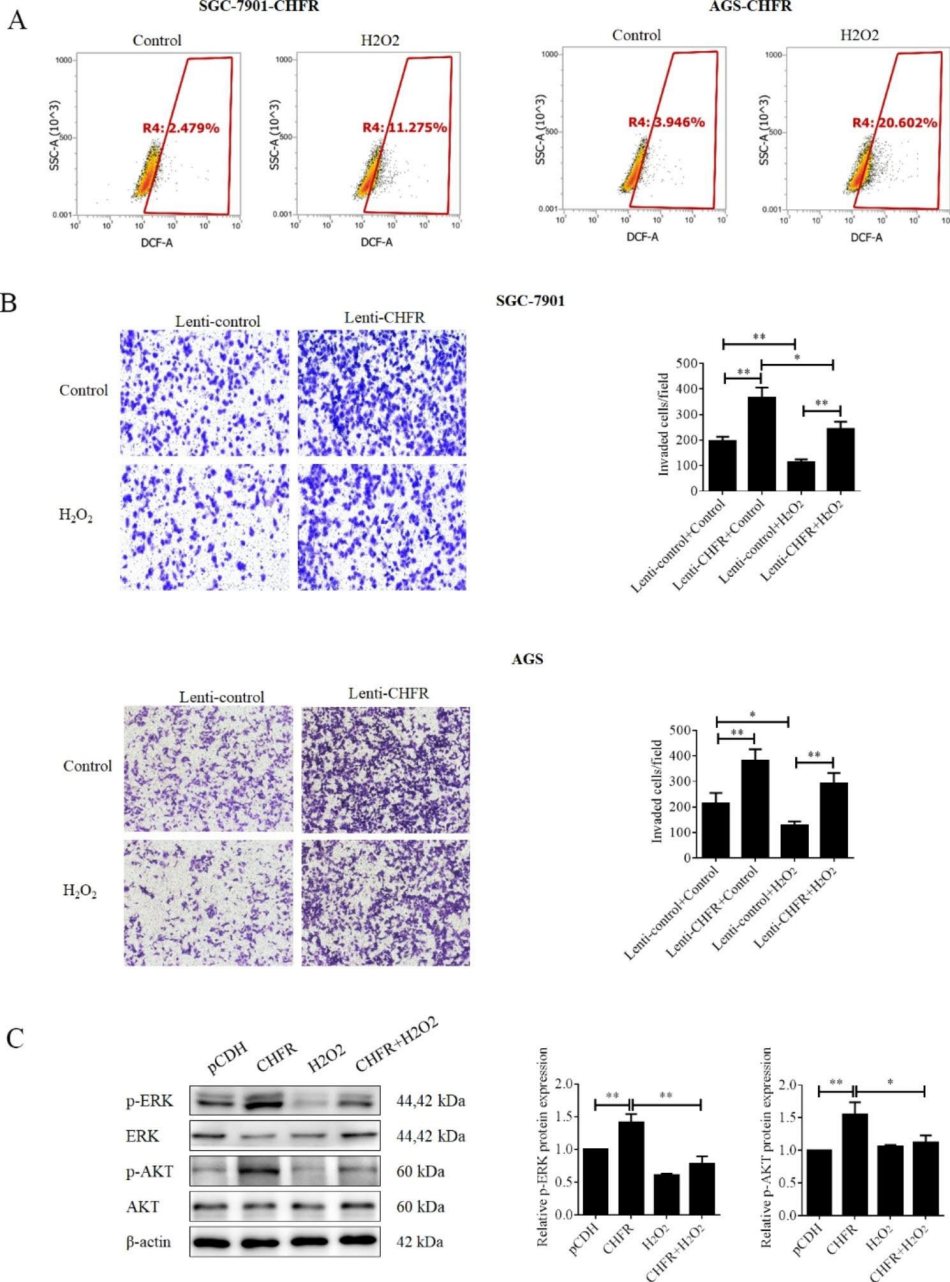
ROS generated by H_2_O_2_ blocked the functions of CHFR in gastric cancer cells. (**A**) CHFR stably expressed SGC-7901 and AGS cell lines were treated with 500 µM H_**2**_O_**2**_ for 1 h. ROS levels were detected by flow cytometry. (**B**) After treatment with H_**2**_O_**2**_, transwell assay were performed to determine the invasion in the CHFR stably expressed SGC-7901 and AGS cell lines. Quantitative analysis of invaded cells was shown in the histogram. (**C**) After treatment with H_**2**_O_**2**_, Western blot was used for the detection the protein on AKT, p-AKT, ERK and p-ERK in the CHFR stably expressed SGC-7901 and AGS cell lines. Quantitative analysis expression of proteins was shown in the histogram. Data was presented by mean ± SD for three separate experiments. *P < 0.05; **P < 0.01

### CHFR activated the phosphorylation of AKT and ERK, and cell invasion of GC cells in a ROS-dependent manner

To further confirm whether CHFR-induced decrease of ROS level contributed to the activation of AKT and ERK, CHFR stably expressed gastric cancer cells SGC-7901 and AGS were treated with H_2_O_2_ and the ROS level was also detected. As shown in Fig. 3A, H_2_O_2_ treatment obviously increased the ROS levels in both stable cell lines. Transwell experiments were used to testify whether ROS could directly influence the cell invasion of GC cells. As shown in Fig. 3B, H_2_O_2_ treatment significantly reversed the stimulative function of CHFR on cell invasion in SGC-7901 and AGS cells. More importantly, H_2_O_2_ treatment effectively attenuated the phosphorylation level of AKT and ERK mediated by CHFR overexpression in SGC-7901 stable cells (Fig. 3C). Taken together, CHFR induced decrease of ROS contributed to the activation of AKT and ERK, and cell movability.

### CHFR functions as a ROS regulator by promoting the nuclear factor erythroid 2-related factor 2 (NRF2) expression in GC cells

**Fig. 4:**
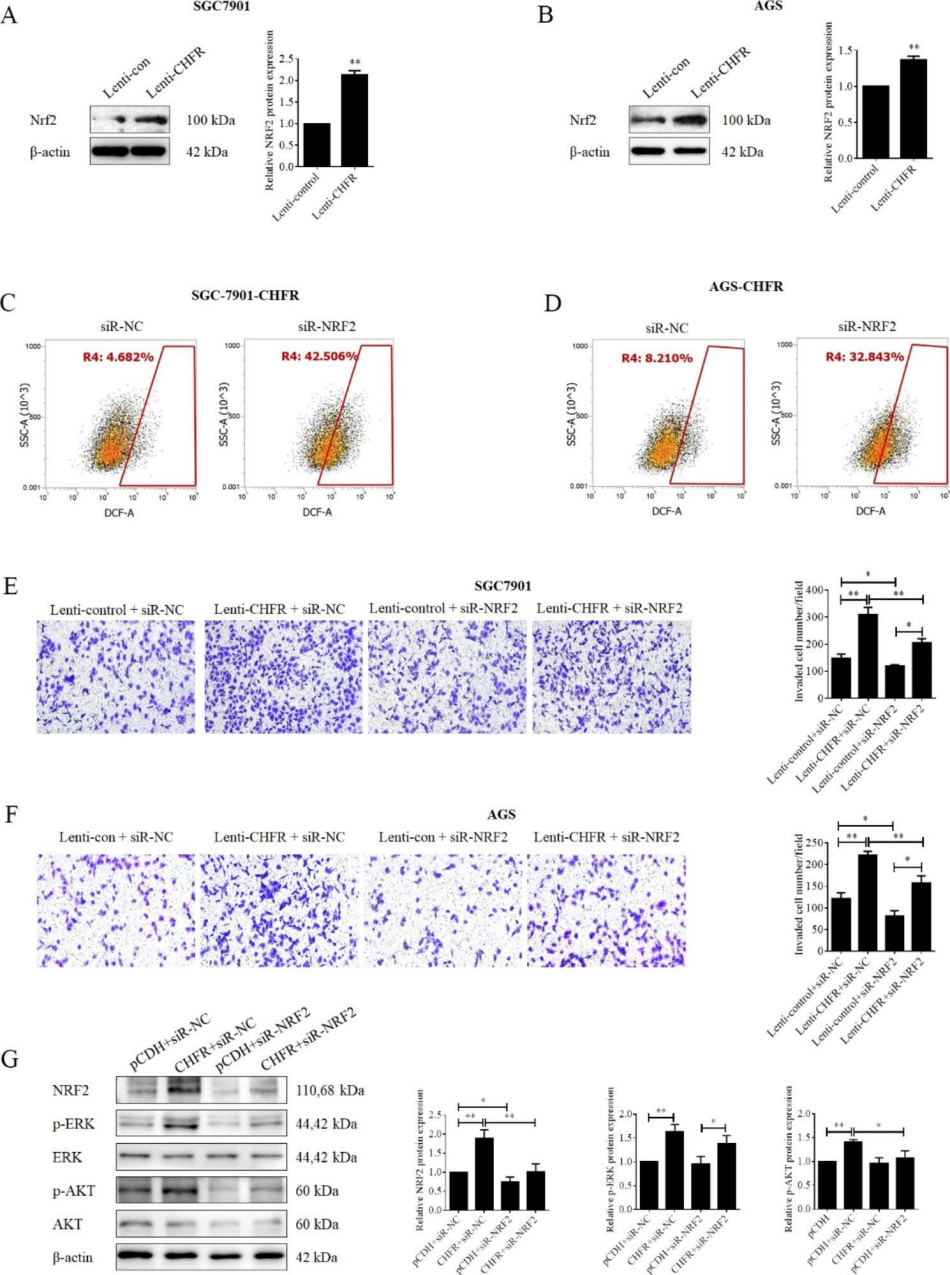
CHFR exerted its functions in gastric cancer cells by promoting the expression of NRF2. (A and B) Western blot was used for the detection the protein expression of NRF2 in the CHFR stably expressed SGC-7901 (**A**) and AGS (**B**) cell lines. Quantitative analysis expression of proteins was shown in the histogram. (C and D) CHFR stably expressed SGC-7901 (**C**) and AGS (**D**) cell lines were transfected NRF2-siRNA or NC for 24 h. ROS levels were detected by flow cytometry. (E and F) CHFR stably expressed SGC-7901 (E) and AGS (**F**) cell lines or control cell lines were transfected NRF2-siRNA or NC for 24 h, transwell assay was performed to determine the cell invasion. Quantitative analysis of invaded cells was shown in the histogram. (**G**) CHFR stably expressed SGC-7901 cell line or control cell were transfected NRF2-siRNA or NC for 24 h, western blot was used for the detection the protein on NRF2, AKT, p-AKT, ERK and p-ERK. Quantitative analysis expression of proteins was shown in the histogram. Data was presented by mean ± SD for three separate experiments. *P < 0.05; **P < 0.01

The excessive accumulation of ROS is an emerging hallmark of cancer. Tumor cells always have an aberrant redox homeostasis to activate onco-signaling and avoids ROS-induced programmed death by orchestrating antioxidant systems. Multifarious modulators are involved in the redox sensing pathways, such as NRF2 [19]. We first examined the role oof CHFR on NRF2 expression, and the data indicated that the overexpression of CHFR significantly enhanced the expression of NRF2 in two stable GC cell lines (Fig. 4A). siRNA was used to knock-down the expression of NRF2 and ROS levels were detected by flowcytometry in two cell lines. As shown in Fig. 4B, the silence of NRF2 effectively elevated the ROS levels in SGC-7901 (Fig. 4C) and AGS (Fig. 4D) stable cell lines. Cell invasion of both two cell lines were also determined by transwell assays, and our results shown that silence of NRF2 not only impaired the cell invasion alone, but also effectively attenuated the role of CHFR overexpression on cell invasion potentials in SGC-7901 (Fig. 4E) and AGS (Fig. 4F) stable cell lines. Finally, the expression of NRF2 and, the activation of AKT and ERK in SGC-7901 stable cells were also examined. As shown in Fig. 4G, CHFR elevated the NRF2 expression and siRNA effectively reversed this trend, and more importantly NRF2 silence effectively attenuated the CHFR-mediated activation of AKT and ERK. All these results suggested CHFR maintained the redox homeostasis by promoting NRF2 expression, and subsequently the activation of AKT and ERK signaling pathways.

### CHFR suppressed the tumor growth and promoted lung metastasis of GC cells in nude mice models

**Fig. 5:**
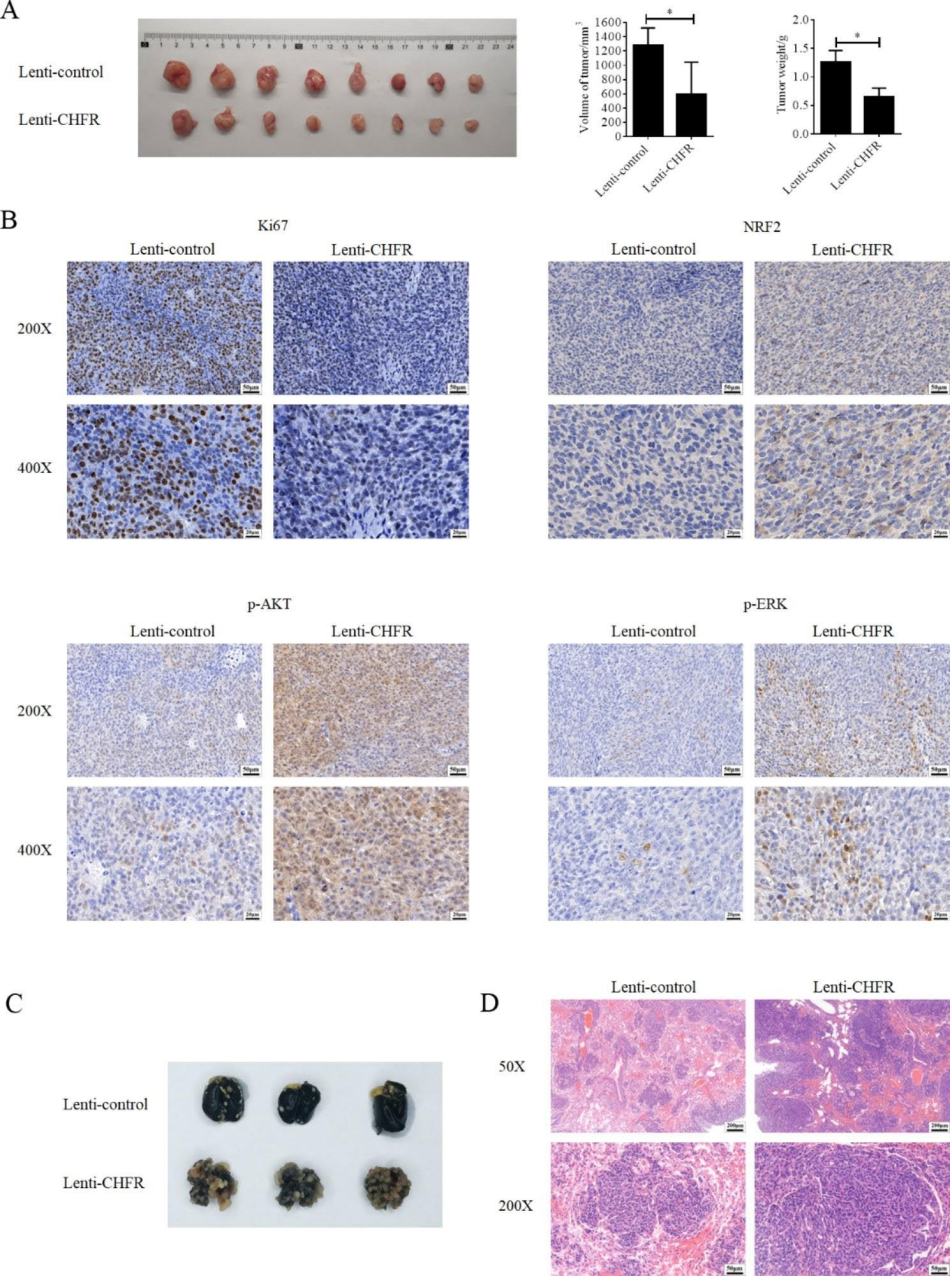
CHFR inhibited the growth of AGS-derived xenograft and enhanced the lung metastasis of AGS cells in nude mice. (**A**) CHFR-stably expressed AGS cells or the control cells were subcutaneous injected into nude mice to establish the xenograft model. The tumor volumes and weights were shown in the left panel. (**B**) IHC experiments were performed to detect the expression of ki67, NRF2, p-AKT and p-ERK. The representative data were shown. (**C**) CHFR-stably expressed AGS cells or the control cells were tail vein injected into nude mice to establish the lung metastasis model. The lungs were shown. (**D**) HE staining was used to determine the metastatic in lung tissue and the representative pictures were shown. *P < 0.05; **P < 0.01

Finally, SGC-7901 stable cells-derived xenografts were obtained using nude mice and our data revealed the ectopic expression of CHFR indeed inhibited the growth of xenografts (Fig. 5A). This finding was consistent with its role on cell cycle progression and also supported by the ki67 expression in tumors. As shown in Fig. 5B, the data of IHC indicated that ki67 expression was obviously low in CHFR overexpressed tumors compared with that in control group. As our data supported that CHFR could enhance the expression of NRF2, IHC assay was also used to detect the expression of NRF2. As shown in Fig. 5B, CHFR overexpressed tumors have a higher NRF2 levels compared with that in control group. Similar expression profiling was observed in p-AKT and p-ERK (Fig. 5B). To validate the role of CHFR on GC metastasis in vivo, lung metastasis model in nude mice was established. Our data revealed that the SGC-7901 cells with CHFR overexpression exerted a significantly higher lung metastasis ability compared with the control group (Fig. 5C). We also chose HE staining experiment to identify the metastasis of GC in lung tissues. As shown in Fig. 5D, more and larger metastasis focuses were observed in lung tissues of CHFR overexpressed group.

## Discussion

As a well-known negative regulator of cell cycle progression, CHFR was regarded as a tumor suppressor gene and hypermethylation in *CHFR* gene promoter were frequently observed in multiple cancer types, such as colorectal cancer and gastric cancer [8, 11]. The methylation level of CHFR has also been proved as a biomarker for predict the prognosis in several cancers [20, 21]. However, some studies also found that lower CHFR expression was independently associated with unfavorable prognosis in acute myeloid malignances [22, 23]. On the other hand, some research proved that downregulation of CHFR could also exerts anti-tumor functions such as sensitizing gastric cancer to PARP inhibitor [23], and impairing the cell migration and invasion potential in gastric cancer cell lines which was reported by our previous study [12]. Therefore, it looks like that CHFR is not an absolute tumor suppressor at least in gastric cancer which was supported by the data from datasets of Kaplan Meier Plotter that CHFR expression was negatively associated with the overall survival rate of gastric cancer patients. However, the underlying mechanisms for the inhibitory roles of CHFR on GC cell migration and invasion remain to be further elucidated.

In our previous study, we found that SGC-7901 and AGS cells expressed relatively lower levels of CHFR among several cancer cell lines, and the metastatic subtype SGC-7901 cells expressed higher level of CHFR compared with primary subtype AGS cells [12]. This results also indicated the CHFR might play a role in GC metastasis. In the current study, CHFR stably expressed GC cells were constructed in these two cell lines to examine the role of CHFR on cell proliferation, cell migration and invasion, and tried to elucidate the possible mechanism. Our data revealed that the ectopic expression of CHFR by lentivirus effectively impaired the cell proliferation rate of SGC-7901 and AGS cells, which was consistent with our previous data under transient overexpression of CHFR[12]. More importantly, the stably overexpression of CHFR indeed significantly enhanced the potential of cell migration and invasion in two GC cell lines. These data implied that CHFR may only acted as a negative regulator of cell proliferation which made it contribute to the cancer development, but probably took part in promoting cancer progression especially cancer metastasis at least in gastric cancer.

Redox homeostasis is extremely essential for the maintenance of diverse cellular processes [24]. Compared with normal cells, cancer cells have higher levels of ROS as a result of it much higher metabolism rate. In fact, the acceleration of accumulative ROS disrupts redox homeostasis and causes severe damage in cancer cells [25]. Unfortunately, the redox balance is maintained due to marked antioxidant capacity in cancer cells [26]. Therefore, to induce oxidative stress by increasing ROS and/or inhibiting antioxidant processes have been a promising strategy for anticancer therapies. Although, an earlier study reported that CHFR could promote SIRT1 degradation under oxidant stress [16], whether CHFR took part in regulating in maintaining the redox homeostasis in cancer was not examined. In this study, the ROS levels in CHFR stably expressed gastric cancer cells and their control cells were compared using flow cytometry. To our surprise, overexpression of CHFR in both SGC-7901 and AGS cells effectively decrease the ROS levels. To our best known, this is the first report that CHFR could influence the ROS generation. Next, the activation of AKT and ERK, both were the key regulator in redox homeostasis [27], were detected in CHFR stably expressed SGC-7901 and AGS cells. Our data presented that the phosphorylation of both AKT and ERK were significantly upregulated. We supposed that CHFR promoted the activation of both AKT and ERK by decreasing ROS in GC cells. To further verified this hypothesis, H_2_O_2_ was used to elevated the ROS accumulation in GC cells. Our data revealed that H_2_O_2_ treatment not only elevated the ROS levels but also reversed the role of CHFR in cell invasion in both CHFR stably expressed SGC-7901 and AGS cells. This finding suggested that CHFR promoted GC cell invasion and activated AKT and ERK activation in a ROS-dependent manner.

Next, we tried to illustrate how CHFR regulated the ROS accumulation in GC cells. NRF2 is a transcription factor which is a member of a small family of basic leucine zipper (bZIP) proteins, and functions as a key regulator for oxidative balance by regulating genes which contain antioxidant response elements (ARE) in their promoters [28]. Therefore, we subsequently examined the expression of NRF2 in GC cell with CHFR overexpressed or not. Our data indicated that NRF2, a key anti-oxidant modulator, was significantly up-regulated by CHFR overexpression in both two cell lines. To further testify whether NRF2 take part in the regulation of ROS levels in GC by CHFR, siRNA against CHFR was obtained to knockdown CHFR expression. As expected, silence of NRF2 not only elevated the ROS levels, but also inactivated AKT and ERK, and attenuated cell invasion of GC cells mediated by CHFR. These findings supported that CHFR decreased the ROS levels in GC cells by promoting NRF2 expression at least partially. Finally, the biological role of CHFR was also assessed in xenograft and lung metastasis models in nude mice. Our data from animal experiments demonstrated that CHFR inhibited tumor growth and ki67 expression, and promoted NRF2 expression, the activation of AKT and ERK, and lung metastasis of GC cells nude mice.

Although increasing studies focus on CHFR and its roles in multiple cancer types including GC were reported. Interestingly, almost all CHFR-related publication in GC concerned the methylation in CHFR promoter and its clinical values [11, 29]. Our results suggested that CHFR was not only acted as a tumor suppressor by inhibiting cell cycle progression, but also promoted the cell migration and invasion by activating AKT and ERK in a NRF2-ROS axis.

## Electronic supplementary material

Below is the link to the electronic supplementary material.

Supplementary Material 1

## Data Availability

The data and materials used in the current study are available from the corresponding author on reasonable request.

## References

[1] Torre LA, Bray F, Siegel RL, Ferlay J, Lortet-Tieulent J, Jemal A (2015). Global cancer statistics, 2012. Cancer J Clin.

[2] Chen W, Zheng R, Baade PD (2016). Cancer statistics in China, 2015. Cancer J Clin.

[3] (2020) Erratum: Global cancer statistics 2018: GLOBOCAN estimates of incidence and mortality worldwide for 36 cancers in 185 countries. CA: a cancer journal for clinicians 70:313. 10.3322/caac.21609 32767693

[4] Jemal A, Bray F, Center MM, Ferlay J, Ward E, Forman D (2011). Global cancer statistics. Cancer J Clin.

[5] Kang D, Chen J, Wong J, Fang G (2002). The checkpoint protein chfr is a ligase that ubiquitinates Plk1 and inhibits Cdc2 at the G2 to M transition. J Cell Biol.

[6] Yu X, Minter-Dykhouse K, Malureanu L (2005). Chfr is required for tumor suppression and Aurora a regulation. Nat Genet.

[7] Kim JM, Cho EN, Kwon YE, Bae SJ, Kim M, Seol JH (2010). CHFR functions as a ubiquitin ligase for HLTF to regulate its stability and functions. Biochem Biophys Res Commun.

[8] Cha Y, Kim SY, Yeo HY (2019). Association of CHFR promoter methylation with treatment outcomes of Irinotecan-Based chemotherapy in metastatic colorectal Cancer. Neoplasia.

[9] Shibata Y, Haruki N, Kuwabara Y (2002). Chfr expression is downregulated by CpG island hypermethylation in esophageal cancer. Carcinogenesis.

[10] Brodie SA, Li G, Brandes JC (2015). Molecular characteristics of non-small cell lung cancer with reduced CHFR expression in the Cancer Genome Atlas (TCGA) project. Respir Med.

[11] Dai D, Zhou B, Xu W, Jin H, Wang X (2019). CHFR promoter hypermethylation is Associated with gastric Cancer and plays a protective role in gastric Cancer process. J Cancer.

[12] Yang S, He F, Dai M, Pan J, Wang J, Ye B (2019). CHFR promotes the migration of human gastric cancer cells by inducing epithelial-to-mesenchymal transition in a HDAC1-dependent manner. Onco Targets Ther.

[13] Lyons AB, Blake SJ, Doherty KV (2013). Flow cytometric analysis of cell division by dilution of CFSE and related dyes. Curr Protoc Cytom Chap.

[14] Pijuan J, Barcelo C, Moreno DF (2019). In vitro cell Migration, Invasion, and adhesion assays: from cell imaging to Data Analysis. Front Cell Dev Biol.

[15] Shu S, Li Z, Liu L (2022). HPV16 E6-Activated OCT4 promotes cervical Cancer Progression by suppressing p53 expression via Co-Repressor NCOR1. Front Oncol.

[16] Kim M, Kwon YE, Song JO, Bae SJ, Seol JH (2016). CHFR negatively regulates SIRT1 activity upon oxidative stress. Sci Rep.

[17] Koundouros N, Poulogiannis G (2018). Phosphoinositide 3-Kinase/Akt signaling and Redox Metabolism in Cancer. Front Oncol.

[18] Rezatabar S, Karimian A, Rameshknia V, et al. RAS/MAPK signaling functions in oxidative stress, DNA damage response and cancer progression. Journal of cellular physiology; 2019. 10.1002/jcp.28334 30811039

[19] Senyuk V, Eskandari N, Jiang Y (2021). Compensatory expression of NRF2-dependent antioxidant genes is required to overcome the lethal effects of Kv11.1 activation in breast cancer cells and PDOs. Redox Biol.

[20] Wang C, Ma W, Wei R (2017). Clinicopathological significance of CHFR methylation in non-small cell lung cancer: a systematic review and meta-analysis. Oncotarget.

[21] Ma K, Cao B, Guo M (2016). The detective, prognostic, and predictive value of DNA methylation in human esophageal squamous cell carcinoma. Clin epigenetics.

[22] Zhou JD, Zhang TJ, Li XX (2018). Methylation-independent CHFR expression is a potential biomarker affecting prognosis in acute myeloid leukemia. J Cell Physiol.

[23] Gao L, Liu F, Zhang H, Sun J, Ma Y (2016). CHFR hypermethylation, a frequent event in acute myeloid leukemia, is independently associated with an adverse outcome. Genes Chromosomes Cancer.

[24] Ursini F, Maiorino M, Forman HJ (2016). Redox homeostasis: the Golden Mean of healthy living. Redox Biol.

[25] Aggarwal V, Tuli HS, Varol A et al. (2019) Role of Reactive Oxygen Species in Cancer Progression: Molecular Mechanisms and Recent Advancements. Biomolecules 9. 10.3390/biom9110735 PMC6920770 31766246

[26] Sznarkowska A, Kostecka A, Meller K, Bielawski KP (2017). Inhibition of cancer antioxidant defense by natural compounds. Oncotarget.

[27] Zhang J, Wang X, Vikash V et al. (2016) ROS and ROS-Mediated Cellular Signaling. Oxid Med Cell Longev 2016:4350965. 10.1155/2016/4350965 PMC4779832 26998193

[28] Ma Q (2013). Role of nrf2 in oxidative stress and toxicity. Annu Rev Pharmacol Toxicol.

[29] Li Y, Yang Y, Lu Y (2015). Predictive value of CHFR and MLH1 methylation in human gastric cancer. Gastric Cancer.

