# Supplementary material for: CHFR promotes metastasis of human gastric carcinoma by activating AKT and ERK via NRF2- ROS axis
Source: BMC Gastroenterol. 2023 Apr 6;23:114. doi: 10.1186/s12876-023-02724-4 (PMC10080934; doi:10.1186/s12876-023-02724-4)
Supplement: Supplementary file 1 — Supplementary Material 1 [file 12876_2023_2724_MOESM1_ESM.pdf]

Figure1 A

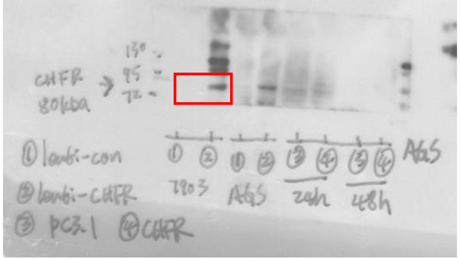

SGC-7901 CHFR

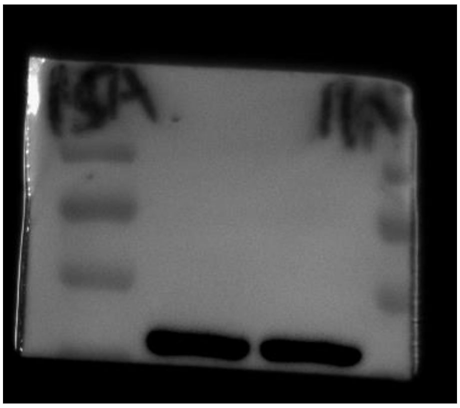

SGC-7901 β-actin

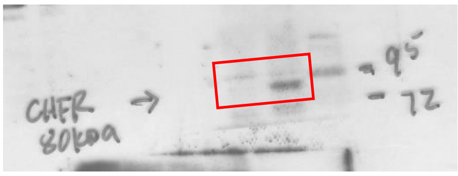

AGS CHFR

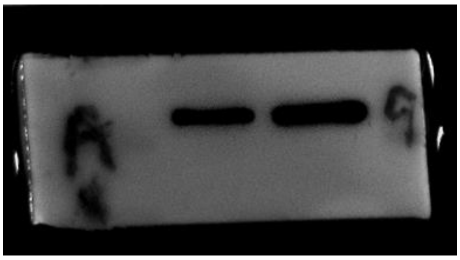

AGS β-actin

Figure2 C

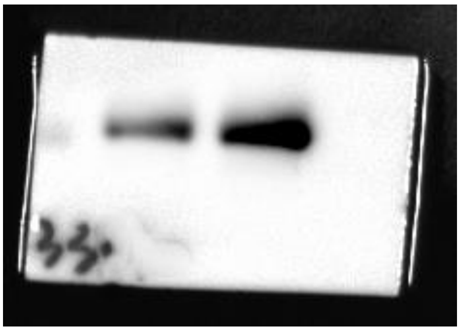

SGC-7901 p-AKT

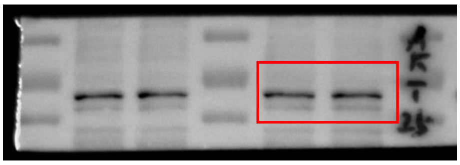

SGC-7901 AKT

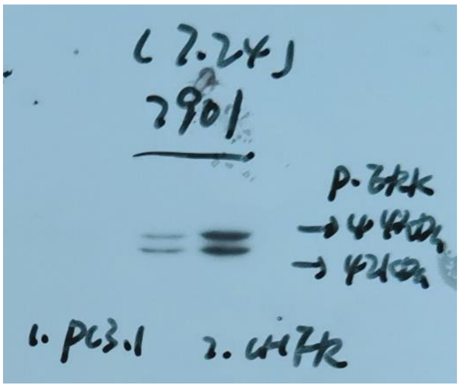

SGC-7901 p-ERK

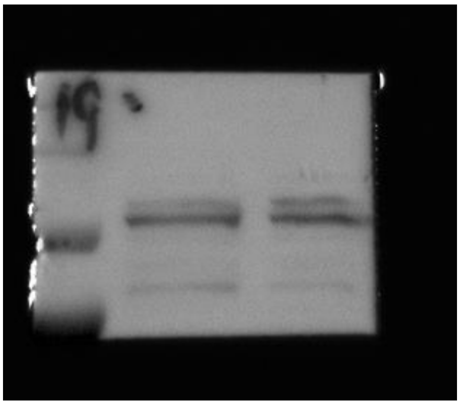

SGC-7901 ERK

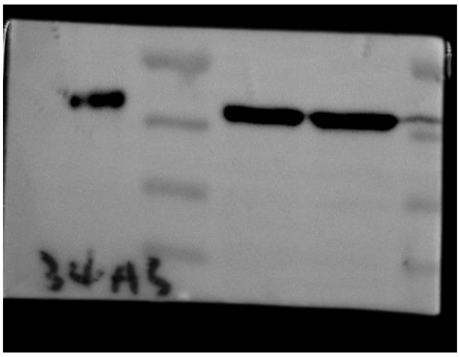

SGC-7901 β-actin

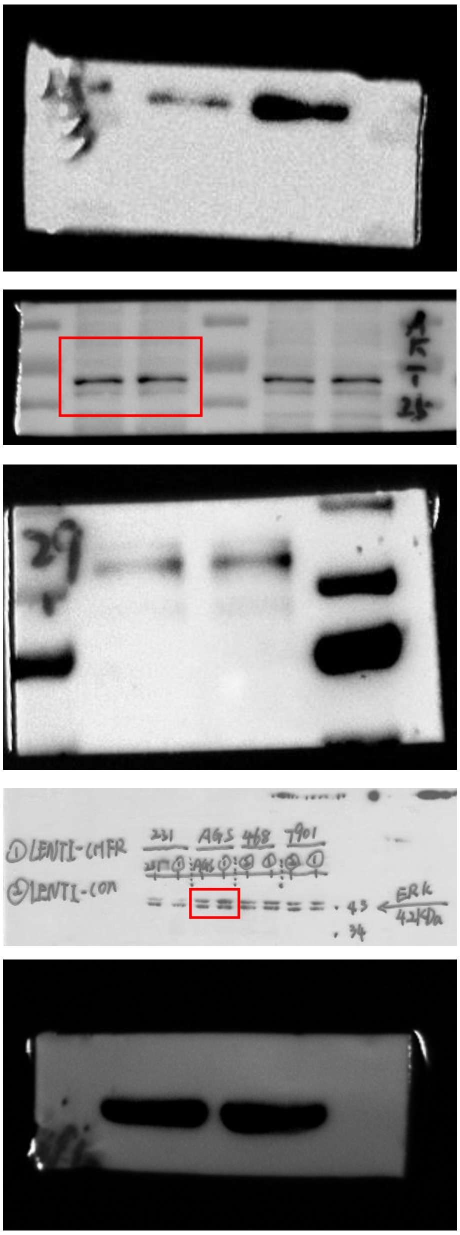

AGS p-AKT

AGS AKT

AGS p-ERK

AGS ERK

AGS β-actin

Figure3 C

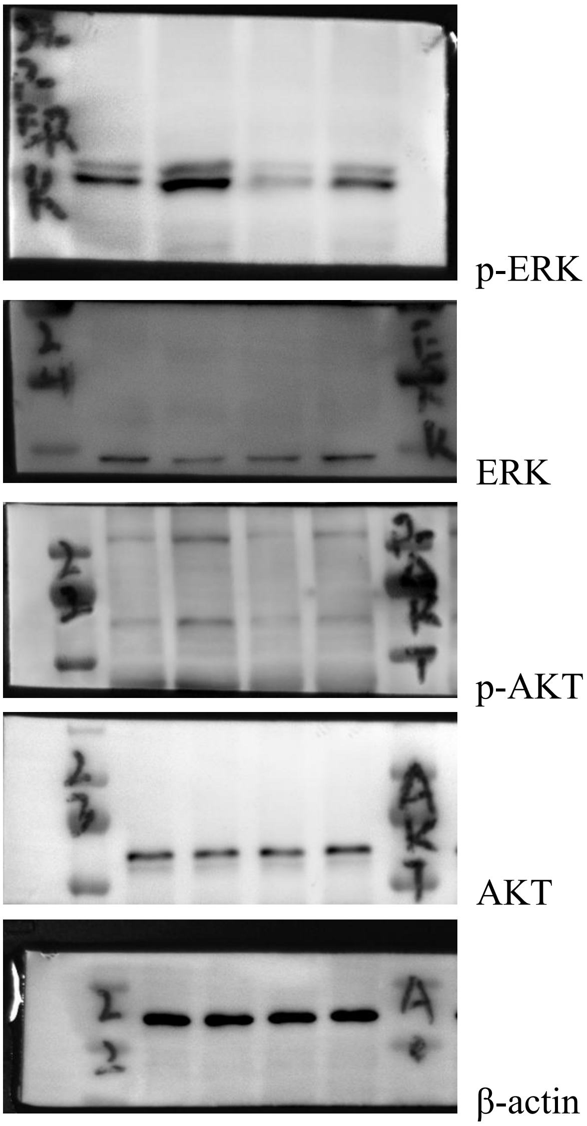

Figure4 G

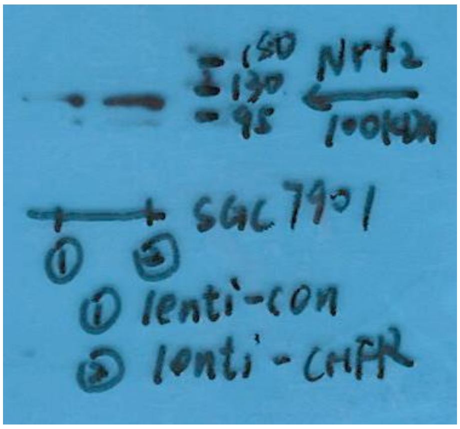

SGC-7901 NRF2

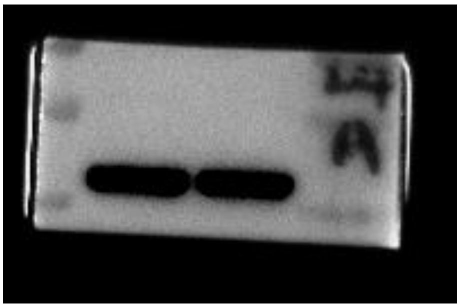
SGC-7901 β-actin

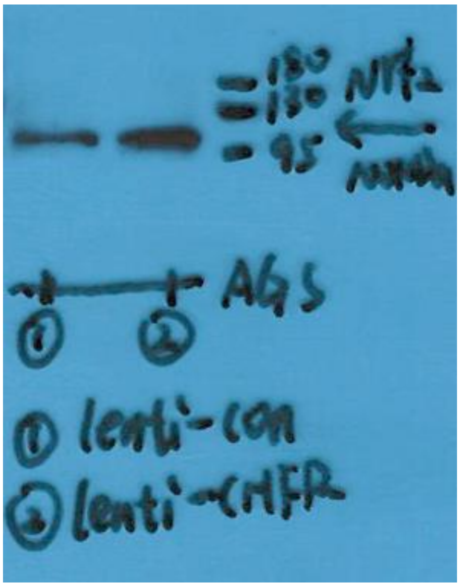

AGS NRF2

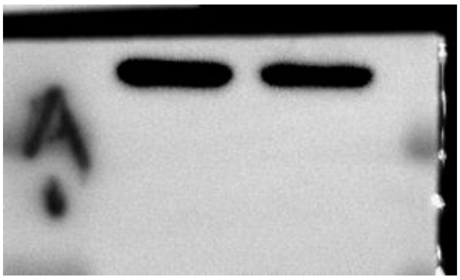
AGS β-actin

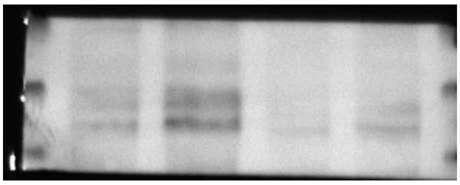
NRF2

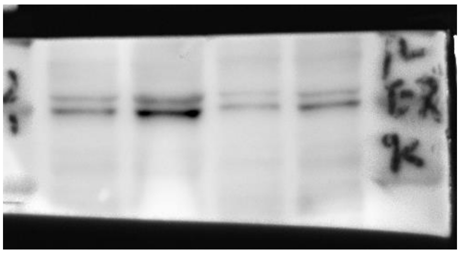
p-ERK

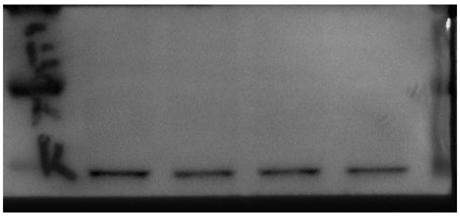
ERK

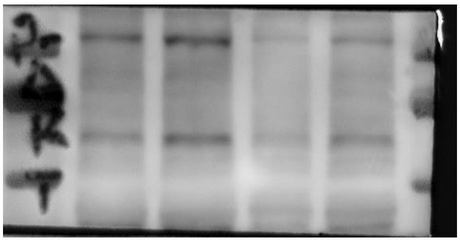
p-AKT

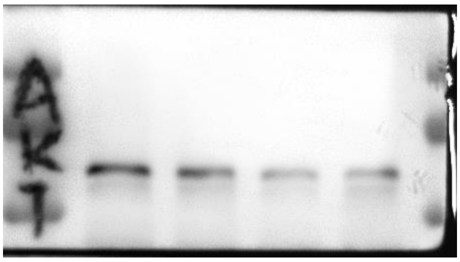
AKT

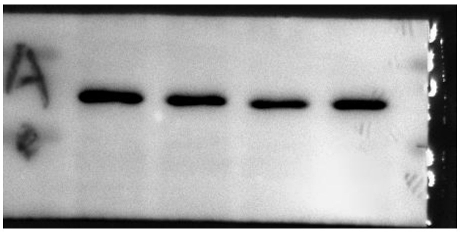
β-actin
